# Trajectories of Development and Socialization of Trans Brazilian Youth Through Self-Portraits

**DOI:** 10.3389/fpsyg.2020.00133

**Published:** 2020-02-07

**Authors:** Elder Cerqueira-Santos, Mariana Valadares Macêdo de Santana, Mozer de Miranda Ramos

**Affiliations:** Department of Psychology, Universidade Federal de Sergipe, São Cristóvão, Brazil

**Keywords:** youth, transsexuality, human development, socialization, childhood

## Abstract

The aim of this research was to explore the meanings of childhood memory established by young people who identify themselves as trans. In a chauvinistic country such as Brazil, sexual socialization of non-cisgender youth is beset by unique challenges, such as discrimination and violence. This study was based on the proposal of a self-portrait method as a content trigger in interviews with three trans young women who reported their life stories. We analyzed data using the oral story technique, through which the described themes came up. The results presented developmental narratives related to gender issue from childhood, which reflected on the experience on youth. Reports of discrimination based on the image of others (family, school, and community) about the gender development of the participants and their relationship with their bodies stand out. We see the importance of reflecting on life history memories in trans identity in order to give meaning to this experience. The influence of heteronormative and sexist Brazilian culture is noticed in the participants’ reports.

## Introduction

The experience of sexuality is a process of interaction between human development and insertion in the social group. Individual and social aspects constantly interfere on human sexual development; even the biological aspects of sexuality must be analyzed in conjunction with the social and cultural aspect (Vandenbosch, 2018). The idea of continuity and inseparability of the sex-gender-desire triad on the sexuality of individuals are established. Based on sexual norms, this ideology proposes a normal system of structuring sexuality based on binary and compulsory heterosexuality (Rich, 1980), which inserts individuals into sexual socialization.

Socialization is a longitudinal process of learning: the behaviors, codes, codes, norms, processes, and many other elements that are shared with individuals enable their insertion in society (Shtarkshall et al., 2007). Environmental stimuli contribute by mediating the individual’s development (Vandenbosch, 2018). It is through this journey that the subject formulates, for example, their sexual scripts and some gender issues. Therefore, sexual socialization is, in general, an intrapsychic, interpersonal and sociocultural process (Gagnon and Simon, 1973); after all, all these different systems are participants in this formulation.

This text describes and reflects on the development and sexual socialization of young transgender people in contemporary Brazil, based on their life history. As a Latin culture, Brazil has a number of myths about the expression of sexuality that span many areas, from the idea of a liberal and permissive sexual culture to the perpetuation of a historical machismo that violates groups and individuals on a daily basis (Fry, 1982; Green, 1999). About two decades ago, the debate in gender studies revolved around the image and objectification of the Safiotti Brazilian woman. Even without the dissolution of this issue (women in Brazil still form a minority and victimized group), other topics arise, especially the issue of homosexuality and prejudice against sexual minorities, including the trans population (Costa et al., 2013). Today, an ultra-conservative, politically and religiously based trend is plaguing the country and affecting the experience of sexuality. We are thinking here of a specific group, non-cisgender young people and their sexual socialization.

In view of this, trans social identity would be the way for people with dissident gender experiences to socio-politically position themselves on behalf of specific demands in the face of cisgenerity (Bagagli, 2016; Caravaca-Morera and Padilha, 2017). Cisgender is the name given to the experience of identifying with the gender attributed by birth. Cisgenerity, as well as compulsory cisgenerity or cisnormativity, is the analytical concepts used to structurally understand the cis experience as a stabilizer of gender norms, just as heterosexuality stands for sexual orientation (Vergueiro, 2013; Bagagli, 2016).

Silva and Oliveira (2015) investigated what they called the process of transsexualization and development. This process “consists of the path toward self-recognition as transsexual and the production of transsexual identity from personal experiences” (p. 485). In their interviews, three important variables in this process are noted, which appear with substantial frequency in the narratives of research in general: childhood plays and games; puberty disguise strategies; and relationships with family and friends. It is difficult to trace elements that would be involved in this process and would be common to all experiences, but the social network supporting the social identity of transgender people appears as an important variable. As discussed, Silva and Cerqueira-Santos (2014) showed that the development of social identity, that is, their awareness of themselves and the groups they belong to, is associated with the ways of thinking body and gender as shared by family, friends, and other subjects from a social network. The trans body is then a volatile, ephemeral, transformable institution that also resides in the field of the nameless in terms of socio-cultural perceptions and sensations (Caravaca-Morera and Padilha, 2017).

Vasco (2015) suggested a different approach to access development memories; she tried to understand what was the relationship that the subjects had with their photographs from childhood and adolescence. There, photography, which could initially be understood only by its indicative character, that is, as the affirmation of a pre-social transition life, appears as a mechanism for elaborating the process of building their gender identities. In qualitative research, the use of photography becomes more complex; the researcher is interested in gaining access to singularities, i.e., access to the subject’s internal world through senses and meanings that encroach on the study subject (Sanches-Justo and Vasconcelos, 2009). Photography appears as a privileged instrument, because in photo-elicitation interviews, i.e., in interviews where there is the presence of photography, answers obtained are emotionally deeper (Croghan et al., 2008). Thus, the process of contemplation of an image is taken into account, “since the image separated from thought becomes a mere residue of the photographed object” (Sanches-Justo and Vasconcelos, 2009, p. 767).

Thus, Volpe (2007) stresses that photography is a way to signify the world, and that it acts as a sort of meeting place for narrative and memory. The aim of this research was to explore the meanings of childhood memory established by young people who identify themselves as trans. In addition, we sought to explore the ties between these memories and the dissident gender experience in youth. In order to do that, we used the self-photographic method, which consisted in the production of photographs and an unstructured interview on this process.

## Materials and Methods

### Participants

Three self-declared young transsexuals participated in this study: I.S., a trans woman, 23 years old; B.P., a trans man, 24 years old; and K.K., a trans man, 22 years old (initials of pseudonimus). The inclusion criteria chosen were trans social identification and over 18. All ethical procedures followed Brazilian law for conducting and publishing research involving human beings. In addition, the institution’s ethics committee approved this project. The consent obtained was both written and informed and it was provided specifically for the publication of indirectly identifiable data.

### Procedures and Instruments

After accepting the survey invitation, six individual meetings were scheduled at times and places convenient to all participants. They were presented with the research, its objectives, the steps and asked about their consent for audio recording. Then the ethical undertaking document was signed and the participant was given a copy.

A digital camera was given to the participants and they were instructed to take pictures that reminded or represented their childhood and adolescence able to “tell significant elements of that time, whether good or bad, and their implications for their gender experience.” They can be abstract photos or of concrete elements, what matters is that they refer to that time. No need to hurry, but I ask you not to take them all at once, like in one afternoon, for example “Think carefully, after all the number is very restricted” (Instruction given to participants).

The deadline for returning the cameras was 1 week, when the researcher contacted the participants and met them to pick the pictures up. Each photograph was printed twice, so that one set would stay with the researcher and be used in the second interview and another set to be given back to the author in a second meeting. This second meeting was scheduled one day after the cameras were handed over, so that they could think about the shooting experience and the content the images referred to. Once again, with their consent, these meetings were recorded.

Like the first time, this meeting was conducted along the lines set by Croghan et al. (2008) for the photo-elicitation interview. The individual interviews lasted about 60 min and were structured informally. Faced with the printed photographs, the participants were invited to talk about each of them in the order that was convenient for them and with little interference from the researcher. The initial statements described the images so that the interviewees would then address more emotionally complex topics such as family and self-image, as foreseen in the method. In their article, the authors describe the method for conducting an interview that uses photographs taken by the subject as triggering elements of speech.

### Data Analysis

The data analysis sought to explore the meanings that each one laid down for their childhood memories, and for that, we based our studies on the formulations of Spink and Medrado (2013) on meaning production. The relationships between memories and the present, the contradictions, formulations of gender, and how all these issues resulted in a sense produced for their own stories were analyzed.

## Results and Discussion

For the three participants, the return to childhood revealed times when certain behaviors were interpreted as displacements of the gender assigned at birth. As part of the process of making sense of their stories, it was necessary for them to build a notion of gender and its relationship with their experience.

The memories of childhood relate to the experience of dissident gender in youth through the uninterrupted process of building the social identity of participants. Recalling the preference of toys, clothes, the way they saw their bodies, as well as the way reference people returned these children’s looks, all were important elements in this process and were symbolized in the photographs. The memories of these childhoods did not return as a painful ghost, but as clues to a potent life. Despite the faults, the forgetfulness, the comments, and the repressions, childhood was remembered as a fertile ground for the search for oneself and for the construction of a life story.

*Because I have my humanity denied, I my humanity was denied. But I also don’t want that to limit my life. I do not mean that my experiences, my life, my childhood were just pain and suffering. So much so that the memories I had, the memories I had, none actually saddened me. It wasn’t something that hurt me, it wasn’t something that pained me. I think there were strong things, difficult things that I had to go through, but that didn’t make me sad. I think I’m happy to have lived that and become who I am today!* (I.S.)

Because they have accepted their trans social identity for longer than K.K., I.S., and B.P. are used to questions such as: *“Who are you? When did you recognize yourself as trans? What does it mean for you to be a woman/man?”* Whether it is for sharing *loci* of militancy and potential visibility with the trans cause, or asking the questions people have asked them every day since transitioning. I.S. comments that society has forced her to think about gender so that people, especially her father, would understand that today she recognizes herself as a woman: *“It was a process where he really needed to see that was really me, that I was sure of that.”*

In this speech from I.S., there are highlights to discuss the relationship established between childhood memory and gender experience in youth. First, on reflecting about gender and its subsequent issues. All the time, I.S. seeks to “denaturalize gender,” understanding it as a social construction: *“.over time, and we have come to understand who we are, but not that we are like this because we were born this way, we are like this because we built ourselves in this way over time.”*

The “denaturalization of gender” referred to by I.S. is the refusal to reduce gender to biological bases, i.e., gender identity would not be a consequence of the genitalia with which the person is born (Lanz, 2014). There is an effort in her speech to understand it from its relational and political character, as a reading of the differences imposed on women and men from their social construction, including questioning the natural kind of the binary paradigm (Scott, 1989; Butler, 2003). From this perspective, the naturalness of gender roles is called into question: men do not cry, they must be strong and providers for the home, while women are sensitive, fragile and devoted to their children. According to Lanz (2014), people suffer from being tied up to these normative ideals.

For I.S., there are models of men and women in society, and people, by “affinity,” recognizes themselves in one of these models or creates their own model. B.P. has an idea of gender more restricted to binaryism, although maintaining a strong criticism of gender stereotypes. These reflections meet the procedural and relational character of social identity, where the process of constitution takes time and occurs uninterruptedly (Silva and Cerqueira-Santos, 2014). Tajfel (1981) states that belonging to one group is established through a relationship with the other, it is by comparison with the other that the subject perceives himself as belonging to a particular group.

To identify oneself as belonging to the “trans world” is to be exposed to regulatory views that reduce dissident experiences to nosological categories. Because of these preconceptions, situations of violence against this population occur on a daily basis, so, as I.S. said, accepting oneself a transvestite or trans is a political act (Bagagli, 2016). Silva and Cerqueira-Santos (2014) argue that a sense of belonging to group in favor of cooperation among members can result in a source of social support. In the first interview, B.P. talks about this process: *“. there is no ‘when you acknowledged yourself,’ there is when you, when population, society finds out who you are, see? You stay there, you live all that in your childhood. You will only put the puzzle together later.”*

Cisnormativity imposes models for men and women and it is based on them that gender roles expected from a child are assigned. It is in the period of childhood that performative utterances are internalized and when stylization of genres takes place (Bento, 2006). The three interviewees recalled moments through the photographs when they were scolded for behaviors that pointed out to a displacement: I.S. recalled how her father regulated the way he walked or the way he supported his hand, while K.K. and B.P. were chided attention, by the way they sat. These are experiences shared by most children, but which gain particular meaning for those who experience a dissident gender experience, as discussed earlier (Kennedy, 2010; Jesus, 2013; Lanz, 2014; Silva and Oliveira, 2015; Vasco, 2015).

The question of this other, who questions them, who reallocates the subject in this place of difference, exposes the relational character of both social identity and gender. I.S. recalled that: *“People kept pointing at me, but I had no idea what it was about. Even though people kept saying: ‘Ah, you’re feminine! You’re a faggot!’ But I didn’t even know what it was to be a fag!”*

The late notion of the social meaning of certain childhood behaviors was shared by the three participants, like a picture you take and only develop years later, during puberty. There is a tension between what is expected/assigned and the behavior of the child, and later the adolescent, who understands what it means to be called “fag” by their peers. Regarding support networks for trans social identity, Silva and Cerqueira-Santos (2014) state that self-awareness (involving aspects such as body and sexual awareness) and about belonging groups (social identity) has a close connection with the way the family, peers, and spouses/boyfriends view these “other” ways of feeling oneself as a woman/man and the gender identity of the subjects in question, since one is not born a subject, but rather becomes a subject (in a brief adaptation of the famous phrase of the philosopher Simone de Beauvoir) from the moment it is possible to perceive oneself as belonging to a certain social and societal reality (Silva and Cerqueira-Santos, 2014).

In I.S.’s narrative, one can notice the fundamental role that the support network, supported mainly by cis and trans women, played in her trajectory. K.K. also acknowledges his mother’s support as a child, just as he talked about his acknowledgment process a few weeks before the first interview. B.P. also finds support in his peers, in his husband and in his family; although inside his mother’s house they do not call him by his name, a fact that justifies the difference due to his parents’ generation.

Regarding self-awareness, which includes aspects of body and sexual awareness, we can highlight the relationship of the external look in the trajectory of the three participants. In I.S.’s childhood, people generally interpreted her games, her way of walking, her desire to have long hair as signs of a *“latent femininity.”* They warned her mother, called her a *“fagot”* at school, cut her off from relationships with other children. According to her, when she was thirteen, puberty comes and with it, masturbation, that’s when she understood why they called her a *“little woman”* and what that meant. She began to think: *“I think I’m gay.”* Even in her early teens, her first boyfriend says he didn’t understand why he felt attracted to her as he didn’t identify himself as gay. The same goes for the lesbian girls she approached. Even if at that point she had not had relations with any man, did not acknowledge herself as a gay teenager, that is how others see her.

When you start taking hormone and wearing Goth makeup, people start to ask, *“who are you?”* Noteworthy are the episodes where she was questioned by her peers in the Candomblé worship place, where she claimed to be androgynous and that is why the men who related to her were heterosexual. Your acknowledgment process is mediated through and by your body, as well as by this outside look, as these are the elements that underlie your understanding of gender and help you build your story.

And then I got to meet gay men and I didn’t identify myself with them. I have started notice “Oh, I’m not like that!” I think differently, I don’t know, I’m not like that. And I identified more with girls, I got closer to girls and I felt that the girls didn’t see that in me, either. That matter I talked about last week, I think this gender issue, I avoid this cliché of saying “oh, my soul is like that or I was born that way.” I don’t think so, I think we build it all. But I think that, for sure, the physical part has nothing to do with it. Because in several periods of my life I noticed that people saw me as a woman, even without my looking as one, looks were irrelevant, see? People felt it, I showed it, even without having to show it by means of my body, appearance, physique, clothing or anything else.

It is also possible to highlight the role of the external look in B.P.’s trajectory. During his childhood, despite constantly playing with his nephew, wearing men’s clothing and preferring elements interpreted as from that world, he did not report direct challenges, as in the case of I.S. This is supposed to be because a boy playing with elements of the female world is interpreted as a more serious displacement than a girl playing with elements of the male world (Welzer-Lang, 2001). Machismo is implicit in this relationship, as B.P. interprets the fact that his mother allowed him to play with his nephew at home, as being more acceptable for him to play with B.P., who *“already acted kind of masculine,”* than to let him play with a girl or the street boys, who disapproved of the behavior.

During his teens, the relationship between his body, the outside look and gender issues intensified for B.P., especially after he started judo training. This is a time that is remembered with joy and as fundamental in his process of acknowledging his gender identity as a trans man. Because he was heavy, there were no girls to fight with or train at school, and that is why his boxing coach placed him with the boys’ group. This recognition of the other differs from the case of I.S., which took place rather in relation to her behavior, while in B.P.’s case it was his body that set him apart. This was also present in the photographs presented by him.

…*at school I always trained running with men. And then, my master always said that I had to have a man’s resistance! So I thought that. “I’m really on the right track!” I just kept looking for the feminine in me. And so, on the one hand I just saw my name. In the name and genitalia. Only! Because as for the rest, I already recognized myself as a boy. So judo, judo was the mark of everything!*

To have a “man’s resistance” means to have something of the male or figuratively to have the strength, the virility of that body. Thus, it was a body that did not correspond to the female stereotype, of a delicate and fragile body, and if it was not female, it was its male opposite. By this time, B.P. was already wearing other male-related cultural signs, such as short hair and short nails. Just like the men’s pants her mother gave her, because the others *“got too frayed.”*

First, we start like this: Being a man is not what I have, what I wear. Yes. But as a result, it helps you to identify yourself. It’s the same, today I am a man and I have breasts. It makes me no less a man, but without my breasts, I’ll feel like the man. It’s as if I was born in the shape I wanted!

Again, a contradiction shows up in B.P.’s speech, where *“it makes me no less a man”* does not seem to relate to *“I’ll feel like the man,”* especially by using the Portuguese male definite article *“o”* (the). Oliveira (2015) points out that among the trans men she researched, there was a type of hierarchy in relation to being more trans based on a comparison of bodies. The more muscle, the more body hair, the better your position relative to other trans men. Although B.P. does not stress this desire to have a body matching the normative standard, it is interesting to note in his speech the power of expressions referring to the male world. Also according to Oliveira (2015), the only body part visible even with clothes that can denounce men (trans) are the breasts; therefore, they are considered as “invaders,” “undesirable” and need to be extirpated.

K.K. recalls when he stopped running in his underwear in the street, commenting on the amazement and at the same time fascination with the idea that his breasts were growing. In much of the interview there was an expression of this feeling of duality about his body, as if K.K. wanted to experience it beyond the norms laid down for the genders. However, when his girlfriend cheated on him with another man in his teens, the issue of genitals comes up markedly for him: *“Why am I not a guy?’ I don’t know. ‘Why don’t I have a dick?’ Because I was not so relaxed at the time, for me men had to have dicks”.* In his speech there is not yet a proper established meaning for these reflections, but there is an attempt to detach himself from the biologizing notion of gender, similar to the efforts of I.S. and B.P. Furthermore, for K.K., the discovery of his body through sexual abuse suffered even as a child further complicates his process of understanding his gender identity. As well as understanding how this process of gender identity recognition fits into the discovery of your still very young body through someone who has abused you more than once.

The body issue is complex and is something that needs to be stressed in the three participants: at the time of the interview, K.K. was experiencing his body from the beginning of hormone therapy, enjoying the first mustache strands that sprouted on his face. I.S. did not take hormones and at no time reported having issues with her body, she even used feminist arguments to build her sense of her body beyond normative standards. B.P. took hormones and yearned for mastectomy surgery, even though his speech showed contradictions.

In general, researches report a question of inadequacy with the body in the speech of people who identify as gender-dissidents (Bento, 2006; Lanz, 2014; Caravaca-Morera and Padilha, 2017). They say things like “I was born in the wrong body” or “I hate my body.” In this research, there were reports of body inscriptions of cultural signs of the genre where one recognizes oneself, whether absence/presence of hair, hair length, clothes, or even medical or surgical interventions. Still, it was not possible to identify the alleged disgust with the body or genitals in particular, beyond B.P.’s comment. The theme or the characteristics of the sample, such as its size or the age of the participants, may be the variables responsible for the matching answers. However, it is still worth stressing those narratives, which establish tension in discourses about the “true” trans experience. As stated by Bento (2006), the narratives point to the diversity of relations with the body and challenge attempts to erase them and inscribe them into categories that homogenize them.

Comparing these narratives with those found by Caravaca-Morera and Padilha (2017), the relationship with the bodies of the participants of this research is close to the talk about the malleability of the bodies. This is related to the possibilities of body transformations, as it they were a blank canvas where you can paint a multitude of watercolors. The gimmicks may not be medications, but there is a construction of this body to meet an expectation of both internal and external gender. Thus, certain reverberations found here also fit the second matrix of the analysis of Caravaca-Morera and Padilha (2017), where the body is understood as its own institution, but regulated and controlled by others. On this point, Rodovalho (2017) says “that in most social interactions there will be no time for you to say what you are, your body having to be able to convey the message as unambiguously as possible” (p. 368).

In the three stories, they recalled moments through the photographs when, during childhood, the body was an instrument of experimentation. Like the hair I.S. wanted to grow long, or in B.P.’s case, the hair that wouldn’t grow long. For K.K., there was sensory experimentation, which he felt when he wore only underwear or when he tied the towel differently on his body. Interestingly, B.P. and K.K. began wearing wider, masculine clothing, prior to their identification as trans men. They both referred to a growing sense of being “comfortable” with those clothes. Just like I.S., who in her teens used hormones before self-identification. This was especially true of B.P., who started wearing two blouses and a coat as soon as her breasts began to grow.

For Rodovalho (2017), self-identification alone is not enough to understand the experience of the trans body; for her, there is always a tense negotiation of meanings between what it means to be and what it means to seem. In general, the three participants talk a little about this issue, as B.P. said:

*We talk a lot that, even though I am a trans man, I can reach the end of my life having everything that a man can get to the point of saying, is that really it? Is something still missing from me? Huh? Because sometimes we seek so much, it may be that I realize myself in every way as a man, I wish I was born a man, and yet I still lack something. Same thing as a child. I could have had it all and say, I was born a boy! And still think that something was missing, think I wish, let’s say, even though I had a ball, a cart, played games, clothes, but I still had to feel that I was missing sneakers. I’m not complete yet, I still have to have male sneakers. Then, that’s it. I think we need characteristics, objects to strengthen us. It’s an indicator, right? Everything in life is an indicator, you have to have an indicator that you’re getting closer, closer. Otherwise, you won’t manage. It’s just like a compass, if you don’t have the direction, if you don’t go in the right direction, it won’t show that your path is stronger, right, right. You’re standing there on the bridge and you don’t know where you’re going, it’s stopped for you. You know what you want, right? That’s how I see it. I always knew what I wanted. I identified myself with trans men, so, I’m a trans man. Okay, if I don’t dress like a man, how will people respect me, know I’m a man? Because we have to see it, despite what we want, we still have a society to face. This is my point of view. I really, really wanted to, so badly, I wish I didn’t need so many masculine characteristics to fit into what a boy is, what a man is. But unfortunately, I live in a society that needs it, even cisgender men need it, they need to wear a beard, they need to wear a men’s outfit, they need to enjoy playing football, and fly kites. So, if they need that, why don’t I need that to recognize myself? But I need an object, of course I need it. Get it?* (B.P., second interview)

The complexity of this relationship with the body and the way it affects the construction of their own story becomes evident in these narratives. The three participants restate the demands society imposes on them to make sure of their gender identity, and that this self-identification must be consistent with the signs that their bodies carry and express. However, even if such body inscriptions do occur, these people are daily exposed to violent reactions from others that will point to something in their bodies or behaviors. Transgender people are denied human dignity. While suffering from the regulation of discourses and incorporating normative cultural signs, the trans body is also a place of resistance production.

… *because people don’t understand that it’s not that I have to boost the female stereotype, but that’s the only thing I find trans, these stereotypes are what I can hold on to in order to show people that I’m female. I don’t need to be a perfect housewife at home, but I can’t get there like this; today I can, because the movement is evolving, but it’s not easy yet, I get here with a beard on my face, and unshaved legs, like feminists claim. They can do that because they are already at another level of debate, we’re still crawling behind. I always said and I will keep saying it: my fight as a transvestite is not to be recognized as a woman, because I think that’s silly, when compared to the fight that I have to move forward, which is to be recognized as a human being!* (I.S., second interview)

Researching the relationship between language and body in the discourses of transgender people, Cassana (2016) demonstrated that despite the sedimentation of senses, language is what also enables the formation of another social body legitimized by the discourse of the transsexual subject. “So, let the body be a celebration party of the impossible” (Cassana, 2016, p. 124).

### Final Considerations

The more classical theories of psychology have for decades maintained that the way young people signify childhood has repercussions on their experiences and experiences in youth and adulthood (Berger, 2011). Developmental Psychology has historically contributed to this discussion by seeing unity in the developmental process from childhood to old age. The chain of experiences contributes to the personality, the formulations about oneself, and the historical integrity of the subjects. There is a rupture in common sense between the passage from childhood to adolescence or during the process of transsexualization; they are redefined under this rationale, adopting a view that aims at seeing the subject as historical and their development as continuous.

The changes these young people experienced during childhood and youth are part of their socialization process (Cerqueira-Santos, 2018). In our sample, gender socialization and sexual orientation were reported as procedural and historicist. Events and experiences seem to be quite integrated into the construction of even the most traumatic identities. This reinforces the view of the comprehensiveness of these subjects (Traverso-Yépez and Pinheiro, 2005). The view of transsexuality signification as a historical breach did not seem evident in this sample. In contrast, trans experiences were linked to the significance of childhood, adolescence and the milestones in the developmental process. Even in the efforts and contradictions to give meaning and connectivity to events.

The specificities of socialization of non-cisgender individuals need to be further investigated. The photography method applied in this study was important in eliciting in these young people a timeline of life history (Croghan et al., 2008). The results demonstrated that this technique is effective for investigating historical events with meaningful integrity. In any case, this technique allows the past to make sense in the present. What was investigated here, therefore, was how non-cisgender young people build and understand their developmental process. And this does not focus on the accuracy of the facts, but on the individualization of these subjects. Transsexuality appeared as an important influential variable in socialization, and consequently, in individuation.

By highlighting the elements that were present in the production of meaning in their stories, the participants consequently clarified the process of building their trans social identity. Reflections about gender and their role in understanding their trajectories stand out. In general, the cis-hetero-normative patterns for the experiments were questioned, resulting in an understanding of the idea of gender identity as a process, something continuous and uninterrupted.

## Data Availability Statement

The raw data supporting the conclusions of this article will be made available by the authors, without undue reservation, to any qualified researcher.

## Ethics Statement

The studies involving human participants were reviewed and approved by the Federal University of Sergipe, Brazil. The patients/participants provided their written informed consent to participate in this study.

## Author Contributions

EC-S was the project general coordinator and academic advisor for the master’s dissertation that led to this study and contributed with the systematization of data and writing of the manuscript. MS was the author of the master’s dissertation that originated this study and was responsible for data gathering and analysis and writing part of the manuscript. MM was responsible for bibliography updating and writing and final proofing of the text.

## Conflict of Interest

The authors declare that the research was conducted in the absence of any commercial or financial relationships that could be construed as a potential conflict of interest.
